# Role of WASP in cell polarity and podosome dynamics of myeloid cells

**DOI:** 10.1016/j.ejcb.2010.05.009

**Published:** 2011-02

**Authors:** James Monypenny, Hsiu-Chuan Chou, Inmaculada Bañón-Rodríguez, Adrian J. Thrasher, Inés M. Antón, Gareth E. Jones, Yolanda Calle

**Affiliations:** a Randall Division of Cell & Molecular Biophysics, King's College London, London SE1 1UL, UK; b Cellular and Molecular Department, Centro Nacional de Biotecnología—CSIC, Madrid 28049, Spain; c Molecular Immunology Unit, Institute of Child Health, University College London, London WC1, UK; d Department of Haemato-oncology, King's College London, London SE5 9NU, UK

**Keywords:** Cell motility, WASP, WIP, Dendritic cell, Podosome, Cell adhesion

## Abstract

The integrin-dependent migration of myeloid cells requires tight coordination between actin-based cell membrane protrusion and integrin-mediated adhesion to form a stable leading edge. Under this mode of migration, polarised myeloid cells including dendritic cells, macrophages and osteoclasts develop podosomes that sustain the extending leading edge. Podosome integrity and dynamics vary in response to changes in the physical and biochemical properties of the cell environment. In the current article we discuss the role of various factors in initiation and stability of podosomes and the roles of the Wiskott Aldrich Syndrome Protein (WASP) in this process. We discuss recent data indicating that in a cellular context WASP is crucial not only for localised actin polymerisation at the leading edge and in podosome cores but also for coordination of integrin clustering and activation during podosome formation and disassembly.

## Introduction

**Fig. 1:**
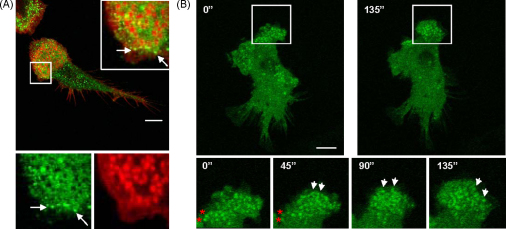
Composition and dynamics of the leading edge in DCs. (A) Confocal micrograph of spleen derived mouse DCs plated on poly-l-lysine coated coverslips overnight. DCs were fixed with 3% paraformaldehyde, permeabilised with 0.05% Triton X-100 and double-stained to detect the distribution of β2 integrin (green) and actin (red). Magnifications of the boxed area show the distribution of β2 integrins and actin separately and merged. β2 Integrins form circular arrays surrounding actin puncta in podosomes and also cluster forming focal contacts at the edge of the leading edge (arrows). (B) Still images of WASP −/− spleen derived mouse DCs expressing eGFP-WASP filmed live using by confocal microscopy taking frames every 15 s. DC were plated on poly-l-lysine coated coverslips overnight and mounted onto viewing chambers. Digits show elapsed time in seconds from the beginning of the film. Magnifications of the boxed area at the bottom of the images show the distribution of eGFP-WASP at the leading edge. eGFP-WASP signal shows the dynamics of podosome assembly and disassembly. White arrows show podosomes that form or mature with respect to the previous time point. Red asterisks point at podosomes that disassemble with respect to the previous time point. As the leading edge extends, podosomes form and mature at the front (white arrows) while at the back of the podosome cluster podosomes disassemble (red asterisks). Bar 10 μm.

As cells migrate they integrate physical and biochemical signals from their environment. Processing of these environmental cues results in cell polarisation through formation of a protruding leading edge and a contractile rear pole that allow for the net translocation of the cell body. Over the past few years, various modes of migration have been described using a range of mechanisms that generate and sustain protrusions at the cell margin (Lammermann et al., 2008; Kardash et al., 2010; Cox et al., 2001). In this article we will confine discussion to integrin-dependent mode of migration of myeloid cells that is required for movement across tissue barriers such as the endothelium and basal lamina of lymphatic and blood vessels. In this type of migration, protrusion of the leading edge is driven by actin polymerisation and sustained by focal complexes generated at the very edge of the leading lamellipodium and by a cluster of podosomes assembled at the interface between the lamellipodium and lamella (Calle et al., 2006a; Linder, 2009) (Fig. 1A). Focal complexes are better known as precursors of the focal contacts seen in many tissue cells cultured in vitro, but podosomes have a limited cellular distribution, being mainly confined to migratory myeloid cells and inducible in endothelial and smooth muscle cells stimulated to take on an invasive motility (Varon et al., 2006; Spinardi et al., 2004). Podosomes are composed of a central cone of branched actin filaments surrounded by a ring of integrins and integrin-associated proteins (Linder, 2009; Calle et al., 2006a) such as vinculin, talin and paxillin. The integrin subfamily recruited to podosomes is cell type specific. In the case of dendritic cells (DCs), integrins of the β1 (van Helden et al., 2006) and β2 families (Calle et al., 2006a) are associated with podosome rings whereas osteoclasts recruit β3 integrins (Chellaiah and Hruska, 2003).

Podosome formation and turnover are fundamental to myeloid cell polarity in this tissue invasive mode of migration. As the leading edges of migrating DCs macrophages and osteoclasts protrude, new podosomes form at the front of an existing cluster to sustain the extending lamellae while podosomes at the back of the cluster disassemble to allow translocation (Fig. 1B, Supplementary video 1). Extension of the leading edge and podosome dynamics require a tight coordination between the actin polymerising and adhesion machineries. As podosomes form, integrins and integrin-associated proteins are recruited to nascent F-actin cores and the process is reversed during podosome disassembly.

## In DCs dynamic podosomes are initiated by chemotactic factors and stabilised by integrin ligands

**Fig. 2:**
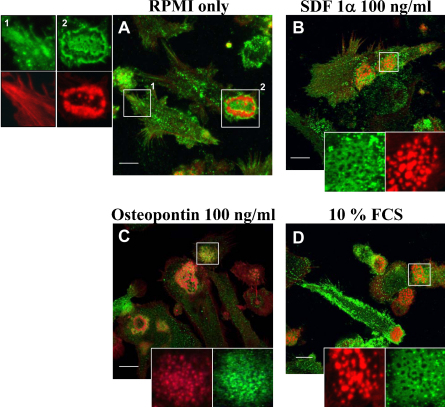
Soluble factors induce DC polarisation and podosome formation. Spleen derived mouse DCs were collected from cultures in RPMI supplemented with 10% FCS, GM-CSF and TGFβ1 and washed 3 times with RPMI to discard supplemented soluble factors. DCs were then resuspended in RPMI (A) or RPMI supplemented with 100 ng/ml SDF1α (B), 100 ng/ml osteopontin (C) or 10% Foetal Calf Serum (FCS) (D) and allowed to spread on poly-l-lysine coated coverslips for 4 h. DCs were fixed with 3% paraformaldehyde, permeabilised with 0.05% Triton X-100 and double-stained to detect the distribution of β2 integrin (green) and actin (red). Images show the merged confocal images of the distribution of both β2 integrin and actin. Magnifications of the boxed areas on the left or at the bottom of the images show the distribution of β2 integrins and actin separately. The majority of DCs plated with RPMI only developed β2 integrin containing focal contacts or rosettes of podosomes (A). SDF1α, osteopontin and FCS promoted cell polarisation (elongated morphology with a distinct leading edge sustained by a cluster of podosomes) and formation of regular clusters of podosomes with the characteristic honeycomb distribution of β2 integrins surrounding actin puncta (B–D). Bar 10 μm.

**Fig. 3:**
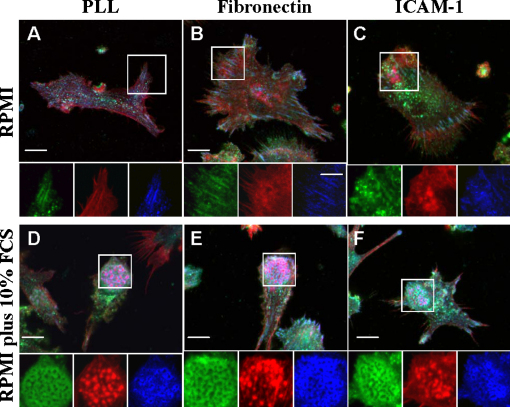
Integrin ligands promote accumulation of integrins and integrin-associated proteins in podosomes. Spleen derived mouse DCs were collected from cultures in RPMI supplemented with 10% FCS, GM-CSF and TGFβ1, washed 3 times with RPMI to discard supplemented soluble factors. DCs were then resuspended in RPMI (A–C) or RPMI supplemented with 10% FCS (D–F) and allowed to spread on poly-l-lysine (10 μg/ml), fibronectin (10 μg/ml) or ICAM-1 (10 μg/ml) coated coverslips for 4 h. DCs were fixed with 3% paraformaldehyde, permeabilised with 0.05% Triton X-100 and co-stained to detect the distribution of β2 integrin (green), actin (red) and vinculin (blue). Images show the merged confocal images of the distribution of β2 integrin, actin and vinculin. Magnifications of the boxed areas at the bottom of the images show the distribution of β2 integrins, actin and vinculin separately. Integrin ligands in the absence of soluble factors failed to induce cell polarisation and formation of podosome clusters behind leading edges. In the presence of soluble factors from FCS, integrin ligands induced accumulation of β2 integrins and vinculin in podosome rings. Bar 10 μm.

During their life span myeloid cells traffic through tissues with different physical and biochemical compositions likely to modulate podosome organisation and dynamics (Cavanagh and von Andrian, 2002). It has recently been shown that the rigidity of the substratum regulates podosome organisation in non-myeloid cells (Collin et al., 2008) as well as myeloid cells (Van et al., 2010) indicating that podosomes can work as mechanosensors. In vitro, both soluble factors present in serum and integrin ligands participate in the regulation of podosome formation and their structural integrity (Calle et al., 2006a; Linder, 2009). DCs plated on poly-l-lysine (a substratum lacking integrin binding sites) or the integrin ligand fibronectin (a ligand for β1 and β2 integrins (Anceriz et al., 2007; van den Berg et al., 2001; Humphries et al., 2006)) in the absence of serum will attach but subsequently fail to form a distinct leading edge or fully assembled podosomes. Instead they mainly assemble β2 integrin containing focal contacts (Figs. 2A and 3A–C). Additionally, between 20% and 30% of the cells develop rings of fused podosome-like structures (Fig. 2A) instead of regularly assembled clusters of discrete podosomes. However, when the serum-free culture medium is supplemented with chemotactic factors such as SDF1α (Vecchi et al., 1999) or osteopontin (Weiss et al., 2001), between 75 and 85% of DCs polarise; forming a leading edge sustained by podosomes similarly to DCs cultured with 10% serum (Fig. 2B–D). These observations indicate that DC chemotactic factors such as SDF1α and osteopontin trigger polarisation and podosome assembly in coordination with the extending leading edge whereas the presence of integrin ligands alone is unable to initiate this process (Fig. 3). We have previously reported that for DCs cultured in the presence of serum, coating the substratum with integrin ligands such as fibronectin or ICAM-1 (the chief ligand for β2 integrins (Humphries et al., 2006)) promotes accumulation of F-actin into podosome cores and recruitment of β2 integrin subunits and vinculin to the podosome ring. Additionally, fibronectin or ICAM-1 promote the stabilisation of podosomes as determined by live interference reflection microscopy (Chou et al., 2006), which correlates with the observed accumulation of podosomal components (Calle et al., 2006a). Taken together, our data support a model of DC migration where chemotactic factors trigger cell polarisation and initiation of podosomes to sustain the leading edge for migration. The presence of integrin ligands from the extracellular matrix or the surface of neighbouring cells promotes maturation and stabilisation of podosomes by inducing further recruitment of structural and regulatory podosomal components. As a result, the leading edge becomes stabilised in the direction of chemoattractant source.

## The Wiskott Aldrich Syndrome Protein (WASP) in myeloid cell polarity and podosome formation

**Fig. 4:**
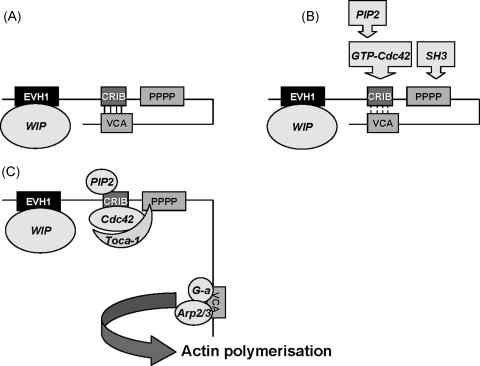
Regulation of WASP activation. EVH1: Ena/Vasp homology 1 domain; CRIB: Cdc42 and Rac interactive domain; pppp: proline rich domains; VCA: verprolin cofilin homology domains/acidic region. (A) In the inactive state, WASP adopts an auto-inhibitory conformation mediated through the interaction between the VCA and the CRIB domains. WIP is bound to the EVH1 domain when WASP is in this conformation. (B) Following cell stimulation, various factors can promote the open conformation of WASP by disturbing the interaction between the VCA and the CRIB domain. These include binding of: (i) GTP-bound Cdc42 to the CRIB domain and (ii) SH3 domain containing proteins (adaptors and kinases) to the proline-rich domain. Given that binding of PIP2 is also required for N-WASP activation and the similarities between these two proteins from the same family, it is likely that PIP2 binding is also required for WASP activation although there is no experimental evidence to support this assumption. (C) Toca-1 binds to both Cdc42 and WIP-WASP complex through the SH3 domain of Toca-1. It is not known if Toca-1 binds directly to WIP, nor experimentally determined whether WIP remains associated with WASP after WASP activation. Once WASP is activated, the VCA domain is released for Arp2/3 activation and actin monomer (G-a) binding, leading to actin polymerisation.

WASP is a member of the WASP/WAVE/Scar family of activators of the actin nucleator Arp 2/3 complex (Takenawa and Suetsugu, 2007). These are adaptor proteins that once activated by the Rho-GTPases Cdc42 or Rac can bind and activate the Arp 2/3 complex as well as recruiting actin monomers leading to actin polymerisation (Takenawa and Suetsugu, 2007) (Fig. 4). WASP expression is restricted to haematopoietic cells and was discovered as the product of the gene that is mutated in the Wiskott Aldrich Syndrome (WAS), an X-linked immunological disorder diagnosed in children (Bosticardo et al., 2009). WAS symptoms include eczema, thrombocytopenia and recurrent infections. Haematopoietic cells from these patients display an abnormal cytoskeletal organisation including the absence of podosomes in myeloid cells (Burns et al., 2004). These cytoskeletal defects translate into impaired leukocyte migration in vitro and in vivo (Burns et al., 2004; de Noronha et al., 2005; Zicha et al., 1998). DCs and macrophages derived from WAS patients or WASP null mice fail to assemble podosomes and form focal contacts instead, independently of substratum composition (Linder et al., 1999; Burns et al., 2001; Calle et al., 2006a). Expression of eGFP-WASP in a WASP null background reconstitutes myeloid cell polarity, podosome formation and chemotaxis (Blundell et al., 2008; Jones et al., 2002; Calle et al., 2006b, 2004). These data indicate that cell polarity and podosome formation induced by chemotactic factors requires WASP. It also suggests that WASP may be a constituent of the podosome initiation complex.

**Fig. 5:**
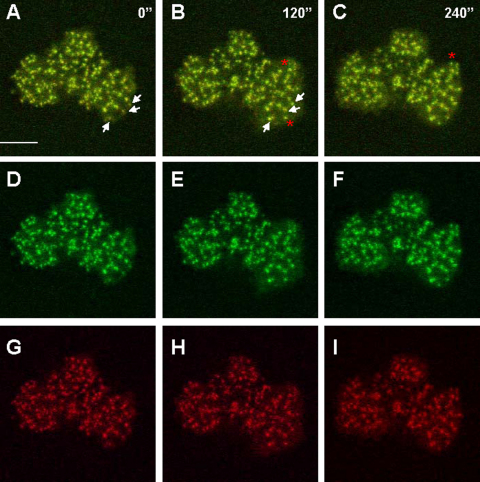
WASP and WIP are recruited simultaneously to nascent podosomes. The monocytic cell line THP-1 was transduced with eGFP-WASP and WIP-mCherry. Clones co-expressing eGFP-WASP and WIP-mCherry were obtained and plated on fibronectin coated coverslips in RPMI supplemented with FCS and 1 ng/ml TGF-β1 overnight and mounted onto viewing chambers. Under these conditions THP-1 cells assemble podosomes similarly to the treatment with TPA (Tsuboi, 2006), which makes them a very useful cellular model to study formation and dynamics of these adhesions. Cells were filmed live using by confocal microscopy taking frames every 15 s. Digits show elapsed time in seconds from the beginning of the film. Yellow colour in panels A–C indicates co-localisation of eGFP (green) in panels D–F and mCherry signals (red) in panels G–I. White arrows point at nascent podosomes in frame taken at the beginning of the film (time 0 s) that increase in size and WASP and WIP content 120 s later. Red asterisks point at newly assembled podosomes with respect to the previous time point. As the leading edge extends WASP and WIP are recruited simultaneously to the core of nascent podosomes suggesting these proteins are constituents of the podosome initiation complex. Bar 10 μm.

WASP interacts at the amino terminus with the WASP Interacting Protein (WIP) (Anton et al., 2007; Ramesh et al., 1997). Like WASP, WIP is also an adapter protein that can interact with various other proteins involved in actin dynamics including WASP and the ubiquitous member of the same family N-WASP as well as cortactin, profilin and actin itself (Anton and Jones, 2006). Acting together WASP and WIP are crucial for the appropriate coordination between cell protrusion and integrin clustering into podosomes during myeloid cell migration. We have shown that WIP regulates the stability and localisation of WASP to podosomes (Chou et al., 2006). WIP null DCs fail to form podosomes and like WASP null DCs, assemble focal contacts instead. In the absence of WASP or WIP, DCs generate random protrusions at the cell margin that rapidly collapse back into the cell body. The focal contacts assembled by these cells allow firm adhesion onto the substratum but they are very stable (Chou et al., 2006; Calle et al., 2006b) and do not form and disassemble in coordination with the protrusion of the cell margin. Additionally, we and others have shown that in the absence of WIP, WASP is extensively degraded by the protease calpain and/or the proteosome (Chou et al., 2006; de la Fuente et al., 2007). However, upregulation of WASP in a WIP null background by inhibiting the activity of calpain does not lead to reconstitution of podosomes. Instead, WASP localises into amorphous aggregates containing actin filaments and integrins organise forming focal contacts (Chou et al., 2006). Overall, our work suggests that WASP and WIP work as a functional unit involved in podosome initiation by coordinating actin polymerisation and integrin configuration. The presence of WASP and WIP in the podosome initiation complex is also supported by our observations using the monocytic cell line THP-1 differentiated into macrophage-like cells by treatment with TGFβ1 and plated on fibronectin. Live cell imaging of THP-1 cells co-expressing eGFP-WASP and WIP-mCherry shows that WASP and WIP are recruited simultaneously to nascent podosomes behind the extending lamellae (Fig. 5, Supplementary video 2).

**Fig. 6:**
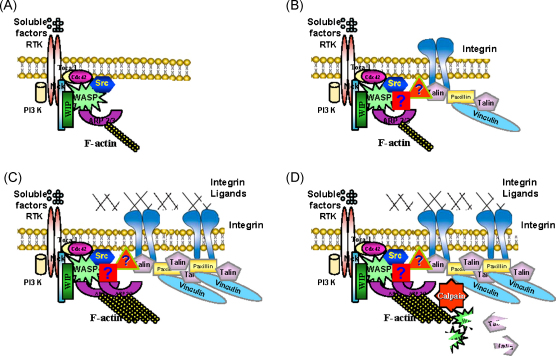
Proposed model of the role of WASP in podosome formation and dissolution. (A) Soluble factors bind to their corresponding receptors which are likely to be receptor tyrosine kinases (RTK) as the ones illustrated in the figure or G-coupled protein receptors, and lead to recruitment and association of PI3K, Cdc42 and WASP and WIP in a complex perhaps linked to other adaptor proteins such as Nck. Additionally, GEFs are recruited leading to activation of Cdc42 and likely Toca 1 is also recruited leading to WASP activation and Arp2/3-mediated actin polymerisation. (B) Simultaneously, integrins are recruited to nascent actin cores in a process where WASP and WIP work as a functional unit to bridge forming actin filaments and integrins leading to formation of the characteristic circular arrays of integrins and integrin-associated proteins surrounding the podosome cores. Other molecules that may provide the link between the WASP-WIP functional unit, F-actin and integrins remain unknown and are symbolised in the diagram with question marks. (C) If integrin ligands are present in the vicinity of the cell, recruited integrins will engage and lead to a feed back loop of activation of PI3K and Cdc42 leading to further WASP activation, actin polymerisation and recruitment of integrins resulting in increased integrity and stabilisation of podosomes. (D) Once podosomes reach a critical size and locate at the back of the cluster, calpain is activated and in parallel WASP and talin (and perhaps other still unidentified podosomal components) may become more sensitive to calpain-mediated cleavage resulting in dissolution of podosomes. Some of the drawings used in these diagrams where obtained from the publication of DeMali and Burridge (2003) with permission from the authors.

Based on our data, we propose a model where myeloid cells in contact with a rigid or semi-rigid substratum respond to chemotactic factors that trigger asymmetric extension of initial protrusions at the cell margin to generate a leading edge. Additionally, chemotactic factors coordinate the initiation of podosomes by activating WASP, which is recruited to the extending protrusion and to adhesion sites in a complex with WIP, leading to actin polymerisation at the leading edge and formation of actin cores in podosomes (Fig. 6A). Integrins are recruited to form a ring around the nascent actin cores and here WASP and WIP also play a key role by generating a molecular platform or adhesome that provides the adequate molecular interactions that link the forming actin filaments and integrins (Fig. 6B). If integrin ligands are present in the environment (extracellular matrix or the surface of neighbouring cells) podosomal integrins engage with their ligands, leading to a signalling feedback loop of activation of actin polymerisation and further clustering of integrins (Fig. 6C). As a result, podosomes mature and become more stable thus maintaining the leading edge oriented towards the source of chemoattractant.

## WASP regulates podosome dynamics

Assembly and maturation of podosomes maintain the extending cell margin, but in order to allow the progression of the leading edge, podosomes need to release their attachment to the substratum and disassemble at the back of the cluster. This process requires cleavage of WASP and talin by the protease calpain (Calle et al., 2006b) (Fig. 6 D). Hence, WASP is involved in both podosome assembly and disassembly. This is further supported by the behaviour of macrophage podosomes of a WAS patient who presented with a point mutation (Ile294Thr) within the regulatory domain of WASP where it binds to the Rho-GTPase Cdc42 (CRIB domain). This mutation led to enhanced actin polymerising activity of WASP and increased number of podosomes assembled by macrophages (Ancliff et al., 2006). These data suggest that this mutation within the CRIB domain destabilises the closed autoinhibited conformation of WASP provided by the intramolecular interaction between the CRIB domain and the carboxy terminus, rendering WASP constitutively active and consequently resulting in actin polymerising activity. Interestingly, podosomes in macrophages from this patient were observed to be extremely dynamic with a high rate of turnover (Ancliff et al., 2006). These results imply that the open conformation of WASP promotes actin polymerisation but it also induces podosome disassembly and adhesion turnover. We infer from these results that the right balance of active/inactive WASP (open/close conformation) is essential to regulate actin polymerisation as well as podosome disassembly. The concept that the same open conformation of WASP leads to both assembly and disassembly of podosomes may be counterintuitive at first. However, it is reasonable to think that for termination of podosomes actin polymerisation and integrin recruitment have to be discontinued and the same constituents of growing podosomes may contribute to the disassembly process. Perhaps the open conformation of WASP allows binding to other partners involved in podosome dynamics and/or it may allow further subsequent post-translational modifications of WASP that may promote cleavage by the protease calpain and subsequent dissolution of podosomes. The specific signalling mechanisms that make active WASP susceptible to cleavage by calpain leading to podosome disassembly remain unknown and need further clarification.

## Conclusions and future challenges

In the past few years many podosomal components have been identified (Linder and Kopp, 2005; Calle et al., 2006a) and taking focal adhesions as our model, new proteins will continue to be found associated with these transient adhesive structures. However, the exact regulation of podosome initiation and dynamics in response to the cell environment factors remains largely undetermined. We show in this report that in the case of myeloid cells, cell polarisation and podosome initiation to sustain the leading edge requires the presence of soluble factors likely chemotactic or growth factors acting through serpentine and receptor tyrosine kinases, whereas integrin ligands promote the recruitment and accumulation of podosomal components and adhesion stability. The exact coordination of the signalling cascades triggered by soluble factors and integrins leading to cell polarisation and podosome formation and dynamics has yet to be determined.

We and others have shown that WASP is essential for myeloid cell polarity during migration as well as a key component and regulator of podosomes (Cammer et al., 2009; Calle et al., 2008; Chellaiah et al., 2007; Linder et al., 1999). Our recent observations indicate that WASP and WIP are part of the podosome initiation complex suggesting that WASP-driven actin polymerisation in the podosome core in response to soluble factors is crucial during podosome formation. Additionally our work indicates that WASP and WIP work as a functional unit that links actin polymerisation leading to persistence of protrusions at the cell margin as well as formation of podosome cores and the configuration and dynamics of integrins and integrin-associated proteins (Chou et al., 2006; Calle et al., 2008; Dovas et al., 2009). This implies a possible role of WASP in regulation of signalling downstream of integrins and possibly in regulation of integrin activity (avidity and/or affinity). Recent reports support the role of WASP in regulation of both inside-out (Stabile et al., 2010) and outside-in (Shcherbina et al., 2010) integrin signalling suggesting that similar mechanisms may take place during podosome formation. Further work is required to determine the signalling mechanisms involved in WASP mediated regulation of integrin organisation and activity. WASP also regulates disassembly of podosomes during the progression of the leading edge (Ancliff et al., 2006; Calle et al., 2006b) and our data suggest that the sustained open conformation of WASP leads to podosome disassembly. The molecular mechanisms that may sustain the open conformation of WASP are likely to regulate calpain-mediated podosome disassembly and need further clarification.

## Appendix A Supplementary data

Video 1 Simultaneous detection of phase, interference reflection (IRM) and fluorescent signals using live confocal microscopy of spleen derived WASP −/− DC expressing eGFP-WASP plated on poly-l-lysine coated glass viewing chambers. Micrographs were taken 10 s apart and displayed at 10 frames per second. DCs formed highly dynamic podosomes that assemble and disassembled continuously behind the leading edge. Podosomes containing eGFP-WASP located in close contact with the substratum as indicated by the IRM signal are formed at the margin of the extending leading edge while podosomes disassemble at the back for the cluster.

Video 2 Live confocal microscopy imaging of THP-1 cells expressing eGFP-WASP and WIP-mCherry plated on fibronectin coated glass viewing chambers in RPMI supplemented with 10% FCS and 1 ng/ml TGFβ1. Micrographs were taken 10 seconds apart and displayed at 10 frames per second. eGFP-WASP and WIP-mCherry are recruited simultaneously to the core of nascent podosomes suggesting that WASP and WIP are components of the podosome initiation complex.
