# Supplementary figures and images for: Role of WASP in cell polarity and podosome dynamics of myeloid cells
Source: Eur J Cell Biol. 2011 Feb;90(2-3):198–204. doi: 10.1016/j.ejcb.2010.05.009 (PMC3037472; doi:10.1016/j.ejcb.2010.05.009)

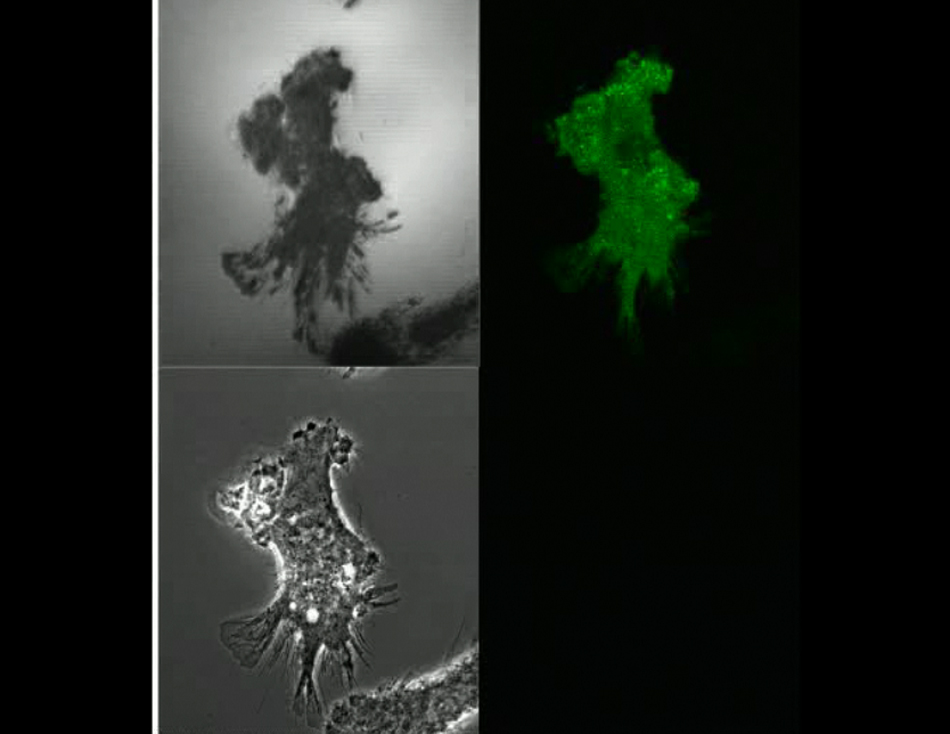

Supplement: Video 1 — Simultaneous detection of phase, interference reflection (IRM) and fluorescent signals using live confocal microscopy of spleen derived WASP −/− DC expressing eGFP-WASP plated on poly-l-lysine coated glass viewing chambers. Micrographs were taken 10 s apart and displayed at 10 frames per second. DCs formed highly dynamic podosomes that assemble and disassembled continuously behind the leading edge. Podosomes containing eGFP-WASP located in close contact with the substratum as indicated by the IRM signal are formed at the margin of the extending leading edge while podosomes disassemble at the back for the cluster. [file mmc1.jpg]

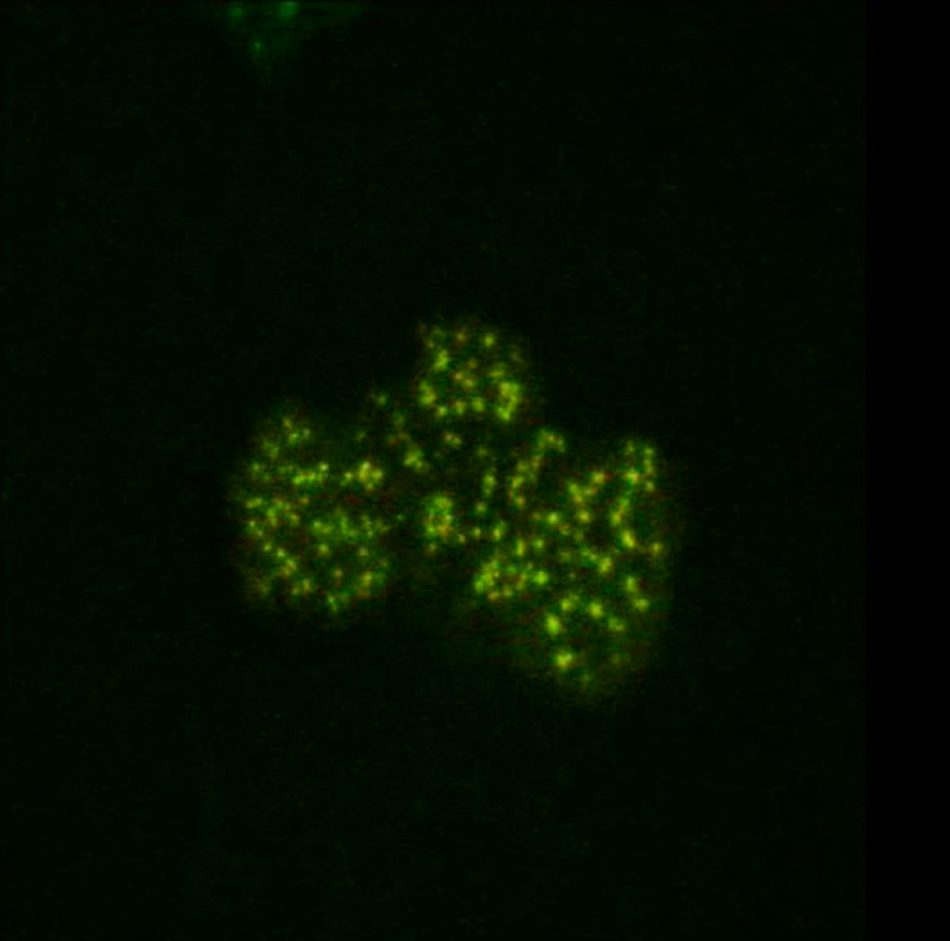

Supplement: Video 2 — Live confocal microscopy imaging of THP-1 cells expressing eGFP-WASP and WIP-mCherry plated on fibronectin coated glass viewing chambers in RPMI supplemented with 10% FCS and 1 ng/ml TGFβ1. Micrographs were taken 10 seconds apart and displayed at 10 frames per second. eGFP-WASP and WIP-mCherry are recruited simultaneously to the core of nascent podosomes suggesting that WASP and WIP are components of the podosome initiation complex. [file mmc2.jpg]
